# Image analysis-based recognition and quantification of grain number per panicle in rice

**DOI:** 10.1186/s13007-019-0510-0

**Published:** 2019-10-31

**Authors:** Wei Wu, Tao Liu, Ping Zhou, Tianle Yang, Chunyan Li, Xiaochun Zhong, Chengming Sun, Shengping Liu, Wenshan Guo

**Affiliations:** 1 Jiangsu Key Laboratory of Crop Genetics and Physiology/Jiangsu Key Laboratory of Crop Cultivation and Physiology, Agricultural College of Yangzhou University, Yangzhou, 225009 China; 2 Jiangsu Co-Innovation Center for Modern Production Technology of Grain Crops, Yangzhou University, Yangzhou, 225009 China; 3 Key Laboratory of Agro-information Services Technology, Ministry of Agriculture, Beijing, 100081 China

**Keywords:** Rice, Grain number per panicle, Image processing, Model, Counting

## Abstract

**Background:**

The number grain per panicle of rice is an important phenotypic trait and a significant index for variety screening and cultivation management. The methods that are currently used to count the number of grains per panicle are manually conducted, making them labor intensive and time consuming. Existing image-based grain counting methods had difficulty in separating overlapped grains.

**Results:**

In this study, we aimed to develop an image analysis-based method to quickly quantify the number of rice grains per panicle. We compared the counting accuracy of several methods among different image acquisition devices and multiple panicle shapes on both *Indica* and *Japonica* subspecies of rice. The linear regression model developed in this study had a grain counting accuracy greater than 96% and 97% for *Japonica* and *Indica* rice, respectively. Moreover, while the deep learning model that we used was more time consuming than the linear regression model, the average counting accuracy was greater than 99%.

**Conclusions:**

We developed a rice grain counting method that accurately counts the number of grains on a detached panicle, and believe this method can be a huge asset for guiding the development of high throughput methods for counting the grain number per panicle in other crops.

## Background

Phenomics involves the gathering of high-dimensional phenotypic data to screen mutants with unique traits and identify the corresponding genes [1]. Current methods for obtaining phenotypic data are generally manual [2], making them time-consuming, labor-intensive, and less accurate. Therefore, such approaches have been impractical for high-throughput measurements during plant growth and development.

The number of rice grains per panicle is a key trait that effects grain cultivation, management, and subsequent yield [3–5], as well as being an important parameter for evaluating the potential of new rice cultivars [6]. Rapid measurement of grain number per panicle could improve the efficiency of scientific research and cultivar development.

Image analysis-based methods have been widely used in many aspects of plant phenotyping. Image-analysis based high-throughput phenotyping platforms have also been applied to measure phenotypic traits of rice, including: plant height, the green leaf area, and rice tiller number [7]. Yang et al. [8] measured the number of panicles on plants using multi-angel color images and an artificial neural network algorithm. The authors reported a reliable, automatic, high-throughput leaf scorer (HLS) for the evaluation of leaf traits, including leaf number, size, shape, and color [9]. Feng et al. [10] developed a hyperspectral imaging system for the accurate prediction of the above-ground biomass of individual rice plants in the visible and near-infrared spectral regions. Zhou et al. [11, 12] used image analysis techniques to assess plant nitrogen and water status. Huang et al. [13] developed a prototype for the automatic measurement of panicle length using dual-cameras, which were equipped with a long-focus lens and a short-focus lens to capture a detailed and complete image of the rice panicle. In addition, image-based methods have been used to characterize seed morphology, including: seeds size, shape, color, and endosperm structure [14–16]. With the advancement of modern optical imaging and automation technology, hardware is no longer a bottleneck for phenotyping. Instead, the analysis and processing of multi-disciplinary optical images have become the new bottleneck [17].

The research on the rapid counting of grain number per panicle has been carried out in different ways. Generally, the panicle is spread out on a white background and held in place by metal pins so that branches and grains are nonoverlapping [14, 18]. It is also an effective way to spread the grains after threshing [16]. These methods are not suitable for rice panicles with severe adhesions in the Yangtze River Basin. Currently, there are two primary methods for the determination of grain number per panicle. The first method is to count the number of grains manually after threshing, which is an incredibly time-consuming and labor intensive process. During the processing of threshed grains, due to the existence of a large amount of awns and overlaid and clustered grains, it is very challenging for traditional algorithms to identify individual rice kernels when they are touching [19, 20]. Husking the grains would make them smoother and easy to separate, but husking also produces broken rice kernels and complicates the counting procedure.

The second methods for determining grain number on each panicle is the most common method and is called on-panicle counting method, which involves counting the number of grains in a spikelet. Collecting an image of the entire panicle is also problematic due to overlaid and clustered grains. To some extent, three-dimensional image acquisition may solve the problem of the touching grains, but equipment to conduct this analyses is expensive and complicated to use.

In this study, we proposed a new counting method that uses image processing and deep learning algorithm to detect rice grain from the image of the primary branch was acquired using digital scanner. Our method would solve grain overlap or clustering problems, be more cost-effective and user-friendly, and facilitate high throughput counting of grain number per panicle in rice.

## Methods

### Field experiment

Since panicle morphology is effected by variety and species, we used two varieties of *Indica* rice (Yangliangyou No. 6 and Fengyouxiangzhan) and two varieties of *Japonica* (Wuyunjing No. 27 and Nanjing No. 9108). In addition, due to the fact that cultivation conditions can affect the grain size and grain number of per panicle, the experiment was organized as two-factor randomized complete block design with seeding density (150, 225, and 300 × 10^4^ plant ha^−1^) and fertilizer (150, 225, and 300 × kg ha^−1^). At the full-heading stage, fifteen panicles per sample group were randomly harvested (Table 1). Total of 540 panicles, which included the spikelet and panicle of different sizes and shapes, were collected in the study.

**Table 1:** Basic information of experimental materials

Density (10^4^ plant ha^−1^)	Fertilizer (kg ha^−1^)	*Indica* rice		*Japonica* rice		Total
		Yangliang you No. 6	Fengyou xiangzhan	Wuyunjing No. 27	Nanjing No. 9108	
150	150	15	15	15	15	60
	225	15	15	15	15	60
	300	15	15	15	15	60
225	150	15	15	15	15	60
	225	15	15	15	15	60
	300	15	15	15	15	60
300	150	15	15	15	15	60
	225	15	15	15	15	60
	300	15	15	15	15	60
Total		135	135	135	135	540

### Image acquisition

**Fig. 1:**
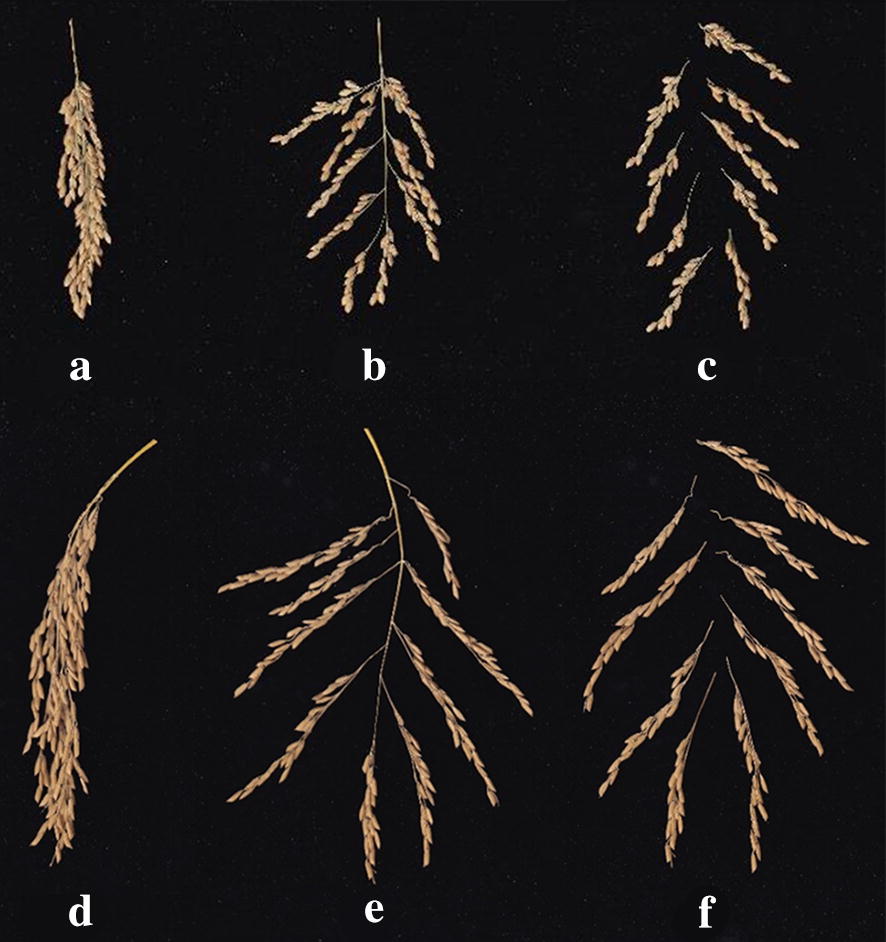
Images of three different panicle shapes acquired using a scanner of the *Japonica* rice and *Indica* rice. **a** *Japonica* rice panicles without manual shaping. **b** The primary branches of *Japonica* rice were manually separated. **c** The primary branches of *Japonica* rice were removed manually. **d** *Indica* rice panicles without manual shaping. **e** The primary branches of *Indica* rice were manually separated. **f** The primary branches of *Indica* rice were removed manually

The different postures of rice panicles have great influences on image recognition. We divided the panicles into three groups based on manual shaping (Fig. 1). Shape A: Panicles were not manually shaped and panicle images were obtained in the natural state. Shape B: the primary branches were manually separated. The moisture content of panicle branch in the mature stage was low and the branch would undergo inert deformation. However, the stem and branch were not completely fractured, and the panicle image was taken in a natural unfolded state. Shape C: the primary branches were removed from panicle stem. The panicle stem and branch were completely fractured, and the panicle image was taken in a natural scattered state. A two-image acquisition method was used for the panicles. The first way is that the samples were placed on a black light-absorbing fabric and the rice panicle images were acquired using digital camera (SONY, model ILCE-6300; equipped with E PZ 16–50 mm, f 3.5–5.6 lens) at a distance of 20 cm above the samples. The image size was 4032 × 3024. The second way is the panicle samples were placed on a scanner (Canon, model LiDE 400, resolution 4800 dpi, and scan speed: color, A4, 300 dpi, 8 s). The scanner was connected to a computer and data were transferred via a high-speed USB2.0 Type-C device. The panicle images were acquired through digital scanner and the image size was 2480 × 3507. The scanner was covered with black light-absorbing fabric to avoid light noise caused by reflection and projection. The basic information of image data was shown in Table 2.

**Table 2:** Basic information of image dataset

Image acquisition method	Panicle shape	*Indica* rice		*Japonica* rice		Total
		Yangliangyou No. 6	Fengyou xiangzhan	Wuyunjing No. 27	Nanjing No. 9108	
Original image data						
Camera	A	45	45	45	45	180
	B	45	45	45	45	180
	C	45	45	45	45	180
Scanner	A	45	45	45	45	180
	B	45	45	45	45	180
	C	45	45	45	45	180
Linear regression training data						
Camera	B	40	40	40	40	160
	C	45	45	45	45	180
Scanner	B	40	40	40	40	160
	C	45	45	45	45	180
Linear regression validation data						
Camera	B	25	25	25	25	100
	C	25	25	25	25	100
Scanner	B	25	25	25	25	100
	C	25	25	25	25	100
Deep learning training and validation data						
Camera	B	5	5	5	5	20
	C	5	5	5	5	20
Scanner	B	10	10	10	10	40
	C	10	10	10	10	40
Deep learning testing data						
Camera	B	25	25	25	25	100
	C	25	25	25	25	100
Scanner	B	25	25	25	25	100
	C	25	25	25	25	100

### Image pre-processing

**Fig. 2:**
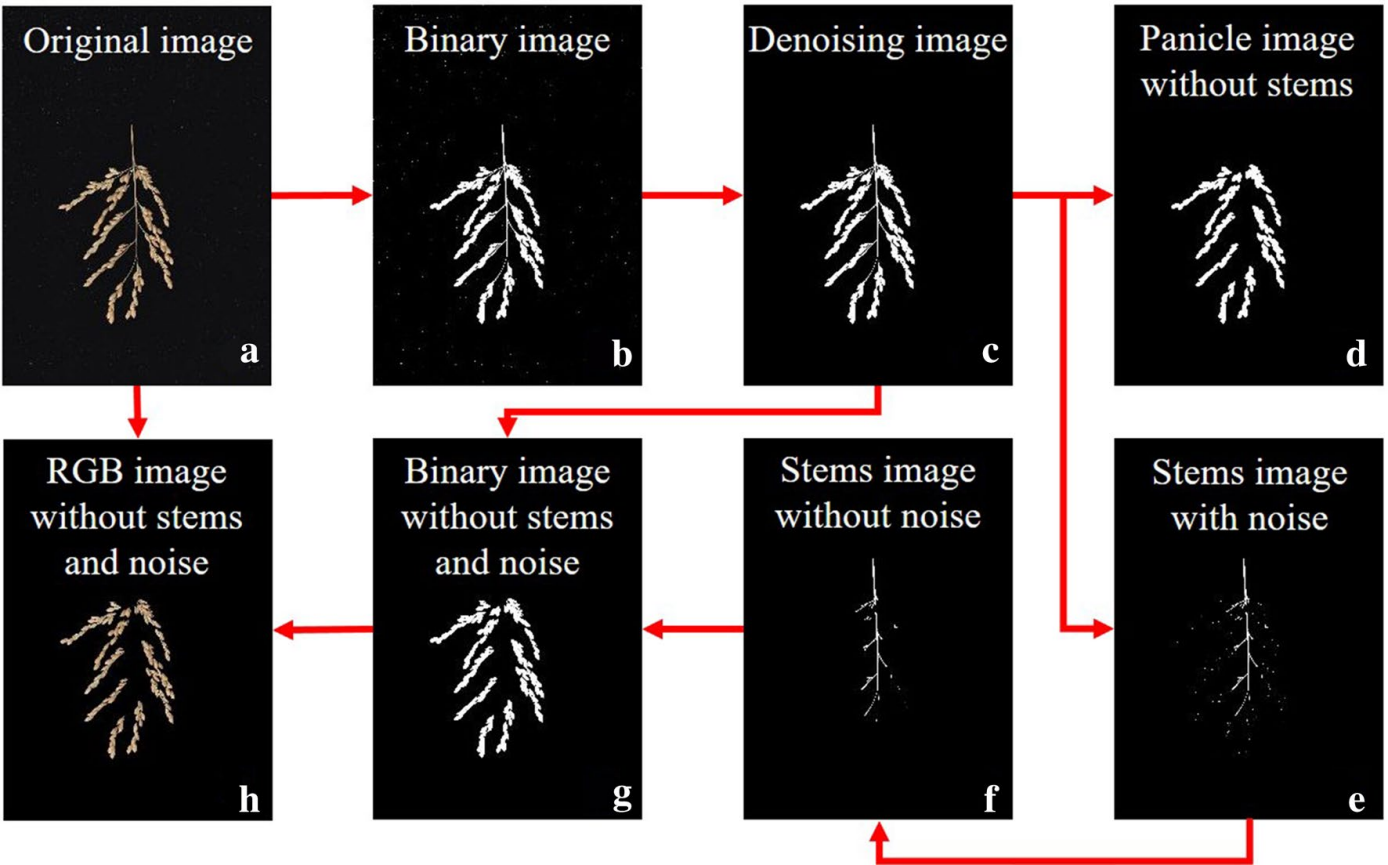
Flow chart of image preprocessing procedures. The scanner-acquired images of *Japonica* rice were used as an example

As shown in Fig. 2a, the contrast between the foreground and background was clear, and the foreground panicles were easily detected using the Otsu Image segmentation algorithm (Fig. 2b) [21]. During image acquisition, black light-absorbing fabric was used to prevent noise caused by light reflection and projection. However, repeated use of same fabric caused contamination by the impurities on the panicle surface and produced some noise. The original image size is 4032 × 3024 or 2480 × 3507, the size of the noise connection area is less than 100 pixels, and the panicle size is more than 100,000 pixels. So, we removed the noise by setting a threshold of 1000-pixels and extracting the connected area (Fig. 2c). The stem of the panicle was not investigated and would be removed. The width of the unfocused stem is 5–10 pixels, only less than half of the width of rice grains (30–50 pixels). Using a 5 × 5 disk mask to erode the denoising image for three times and then dilating it for three times (Fig. 2d). The branch image (without stem) (Fig. 2d) was subtracted from the denoised binary image (Fig. 2c) to obtain a stem image with noise (Fig. 2e) which was generated due to the excessive operation of eroding panicles in the previous step. Similarly, we removed the noise by setting a 200-pixels threshold and extracting the connected area (Fig. 2f). Next, the stem image (without noise) (Fig. 2f) was subtracted from the denoised binary image (Fig. 2c) to obtain a binary image without the stem or noise (Fig. 2g). The RGB image without stems or noise (Fig. 2h) were used for further image processing.

### Algorithm for the calculation of grain number per panicle

For untouched grains, the general counting method is to calculate the number of connected regions in binary images. When it touched, several methods were used to split the clustered kernels, including: dilation and erosion operation, the watershed method, corner detection, and feature matching. However, each method has its limitations in our study, which will be discussed in detail later in this paper. We designed two methods as follows.

#### Linear regression algorithm

**Fig. 3:**
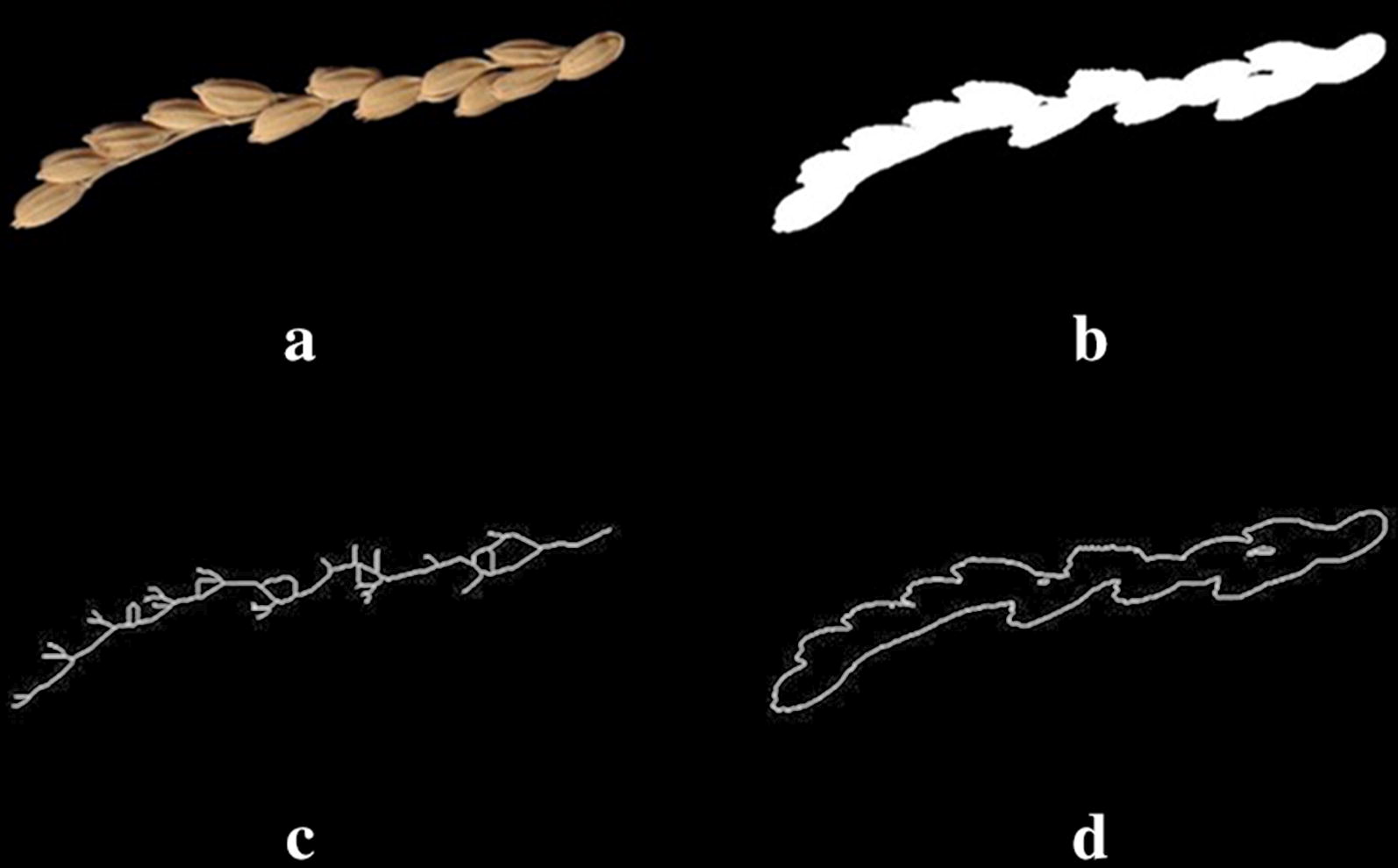
Parameter extraction of *CD*, *Sk* and *Co*. **a** Original image of branches. **b** Extraction of *CD* parameter. **c** Extraction of *Sk* parameter. **d** Extraction of *Co* parameter

Coverage, corner point, etc. are effective feature parameters in image processing and analysis [22]. In this study, three parameters were used as candidates for the construction of linear regression models to count the number of grains per panicle, including: coverage degree (*CD*), skeleton (*Sk*), and contour (*Co*). The result of each parameters extraction is shown in Fig. 3.

The parameter of all primary branch was first extracted from each panicle images. The sum of each branch parameter was used as the entire panicle parameter. All three parameters using the following equation:

1 $$ CD = \frac{{N_{cd} }}{{S_{im} }} $$

2 $$ Sk = \frac{{N_{sk} }}{{S_{im} }} $$

3 $$ Co = \frac{{N_{co} }}{{S_{im} }} $$

where *S*_*im*_ is the size of original image (Fig. 2a); *N*_*cd*_ is the number of pixels with value of 1 in coverage image (Fig. 3b); *N*_*sk*_ is the number of pixels with value of 1 in skeleton image (Fig. 3c); and *N*_*co*_ is the number of pixels with value of 1 in contour image (Fig. 3d).

The parameters were normalized using min–max normalization method as shown in the following equation:

4 $$ CD^{{\prime }} = \frac{{CD - CD_{min} }}{{CD_{max} - CD_{min} }} $$

5 $$ Sk^{{\prime }} = \frac{{Sk - Sk_{min} }}{{Sk_{max} - Sk_{min} }} $$

6 $$ Co^{{\prime }} = \frac{{Co - Co_{min} }}{{Co_{max} - Co_{min} }} $$

where *CDʹ* is the normalized *CD*, *CD*_*min*_ is the minimum value of *CD*, *CD*_*max*_ is the maximum value of *CD*, *Skʹ* is the normalized *Sk*, *Sk*_*min*_ is the minimum value of *Sk*, *Sk*_*max*_ is the maximum value of Sk, *Coʹ* is the normalized *Co*, *Co*_*min*_ is the minimum value of *Co*, *Co*_*max*_ is the maximum value of *Co*.

Since the actual measurement was an integer and the model-predicted number was not always an integer, we rounded predicted number to integers for comparison. Table 2 is the image information used for regression model training and verification. In order to increase the sample size, the 45 shape A panicles were processed into 20 shape B panicles and 30 shape C panicles to acquire images again. The constructed model was evaluated using *R*^2^ and *RMSE*.

#### Deep learning algorithm

**Fig. 4:**
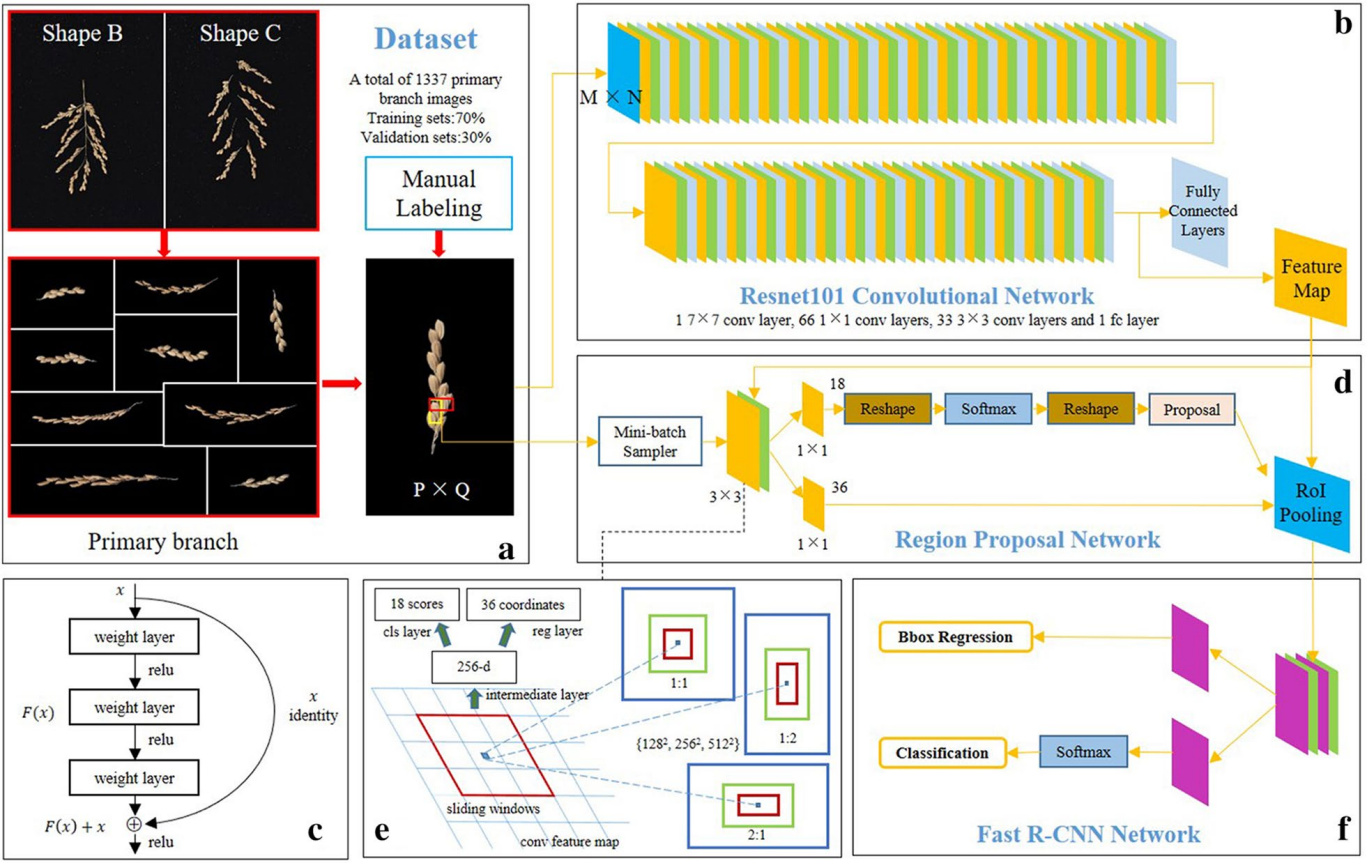
The flowchart of Deep Learning method. **a** Dataset. **b** Resnet101 convolutional network. **c** Residual learning: a building block. **d** Region proposal network (RPN). **e** RPN principle. **f** Fast RCNN network

The second method used in this study was the deep learning method (Fig. 4), which is popular and offers high accuracy performance. In this study, the superior Faster RCNN + ResNet101 network was used for grain identification [23, 24]. Due to the heavy manual labeling work, only 120 original images were randomly selected for the model training and validation (Table 2). After preprocessing, images containing multiple branches and stems were separated into images containing a single branch, and saved as a new image. Total of 1337 images were obtained (Fig. 4a). These images were labeled manually and are available from the author. We divided 70% of the dataset into training sets and 30% of them into validation sets. 400 original images were used for testing. Specifically, an original image was segmented into sub-images. The sub-images were separately identified and then aggregated into test results for one image. The images were sent to the Resnet101 feature extraction network (Fig. 4b) to generate the feature map. The authors of ResNet101 proposed a residual structure (Fig. 4c) to resolve the degradation problem. The selective search (SS) was replaced by region proposal network (RPN) (Fig. 4d). The RPN considers nine possible reference windows (Fig. 4e) at each sliding window (SW) position which can improve the speed and accuracy of object extraction. Finally, training and validation progress is performed by minimizing classification loss and box regression loss. Detailed information about the hardware, software, and model hyperparameters are provided in Table 3. The other hyperparameters were consistent with the original research [24].

**Table 3:** The hardware, software, and hyperparameters configurations for the deep learning model

Project	Content
CPU	Intel Xeon E5-2682v4
RAM	16 G
GPU	Nvidia Tesla P4
Operating system	Ubuntu 16.04 LTS
Cuda	Cuda8.0 with Cudnn v6
Data processing	Python2.7, OpenCV, LabelImg, etc.
Deep learning framework	TensorFlow
Deep learning algorithm	Faster RCNN ResNet101
Num classes	2 (*Japonica* rice grain and *Indica* rice grain)
Batch size	1
Initial learning rate	0.0003
Learning rate	0.0003
Iteration steps	30,000
Minimum confidence	0.9

## Results and analysis

### Comparison on image manually counting method

We compared the counting accuracy of image manual counting methods on two image acquisition ways, two different species of rice and three manually shaped panicles. The ground truth (GT) is panicle grain manual counting. We found that regardless of the image acquisition method, for Shape A, the manual counting accuracy remained below 80%, and was the lowest one of the rice shapes (Table 4). The results indicated that Shape A was not useful because it led to low panicle grain counting accuracy. The maximum counting accuracy for Shape A was 80%, even with the most potent and optimized algorithm. In addition, a 95% counting accuracy could be achieved in the digital camera acquired images, which was at least 3% lower than those in scanner acquired images. This is primary due to the grain distribution on the panicle, which is not even and there being a high quantity of touching grains that are spread out during scanning. The counting accuracies for Shape B and Shape C were both greater than 95%.

**Table 4:** The accuracy of image manual counting for different groups

Image acquisition method	Rice subspecies	Panicle shape	Number of images measured	Accuracy (%)
Scanner	*Japonica* rice	A	100	75.83
		B	100	98.33
		C	100	98.39
	*Indica* rice	A	100	68.46
		B	100	95.34
		C	100	97.51
Camera	*Japonica* rice	A	100	68.08
		B	100	93.35
		C	100	95.26
	*Indica* rice	A	100	66.31
		B	100	89.01
		C	100	93.93

### Linear model analysis

**Fig. 5:**
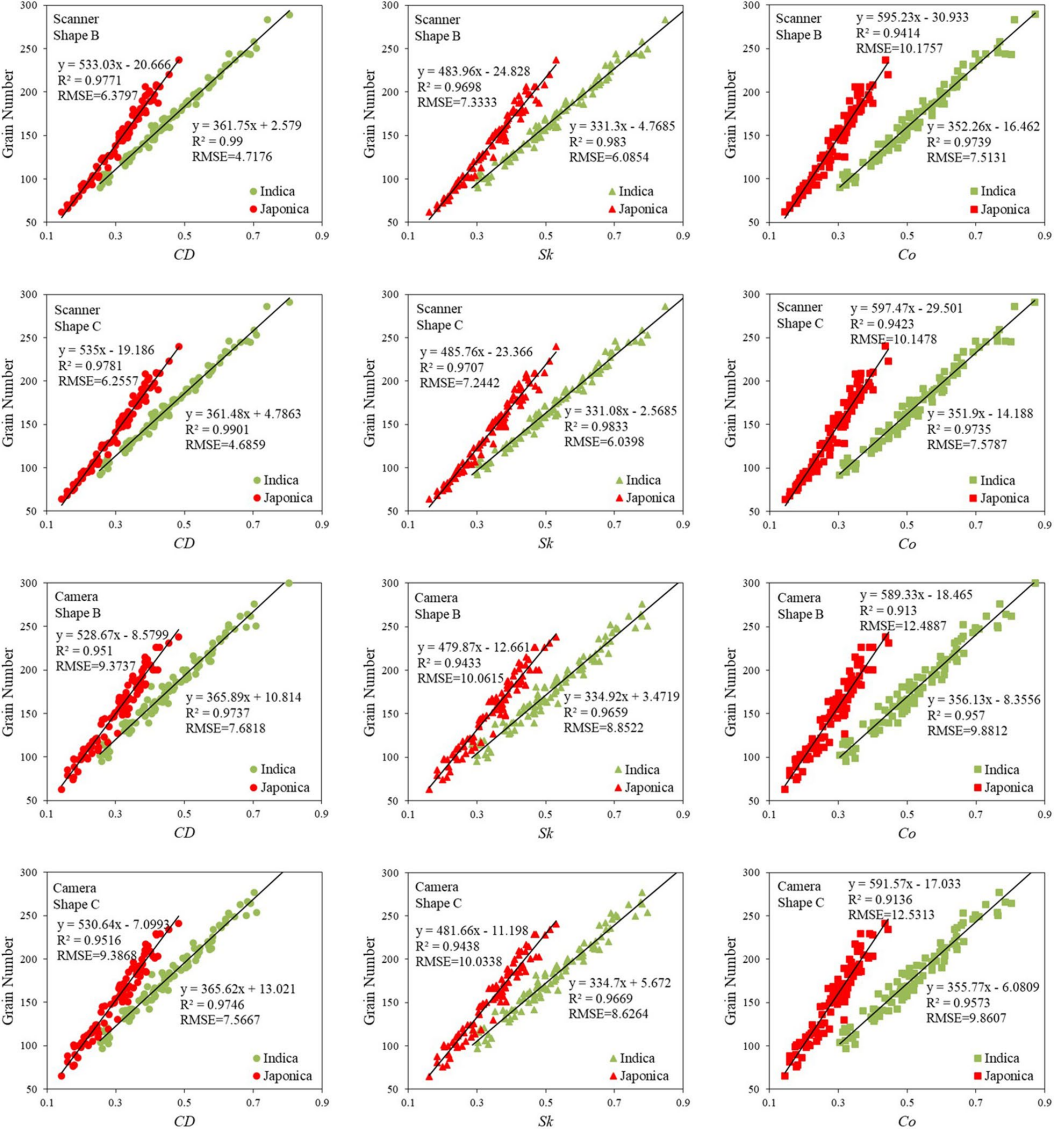
The linear regression model analysis of images with different panicle shapes, and images acquired using scanner and camera

We established 3 univariate linear regression models for counting the grain number on each panicle using *CDʹ*, *Skʹ*, and *Coʹ*. We also compared these 3 models in images that contained different panicle shape manipulation and were acquired using different digital devices. As shown in Fig. 5, for all three models, the *R*^2^ was > 0.90. The *CDʹ*-based linear regression model had an *R*^2^ > 0.95, and had a better performance than *Skʹ*- or *Coʹ*-based models. Overall, images that were acquired from a scanner had lower *RMSE* than images acquired form a digital camera. Furthermore, the RMSE of Shape C + *Indica* rice images was lower than that of Shape B + *Japonica* rice images.

**Fig. 6:**
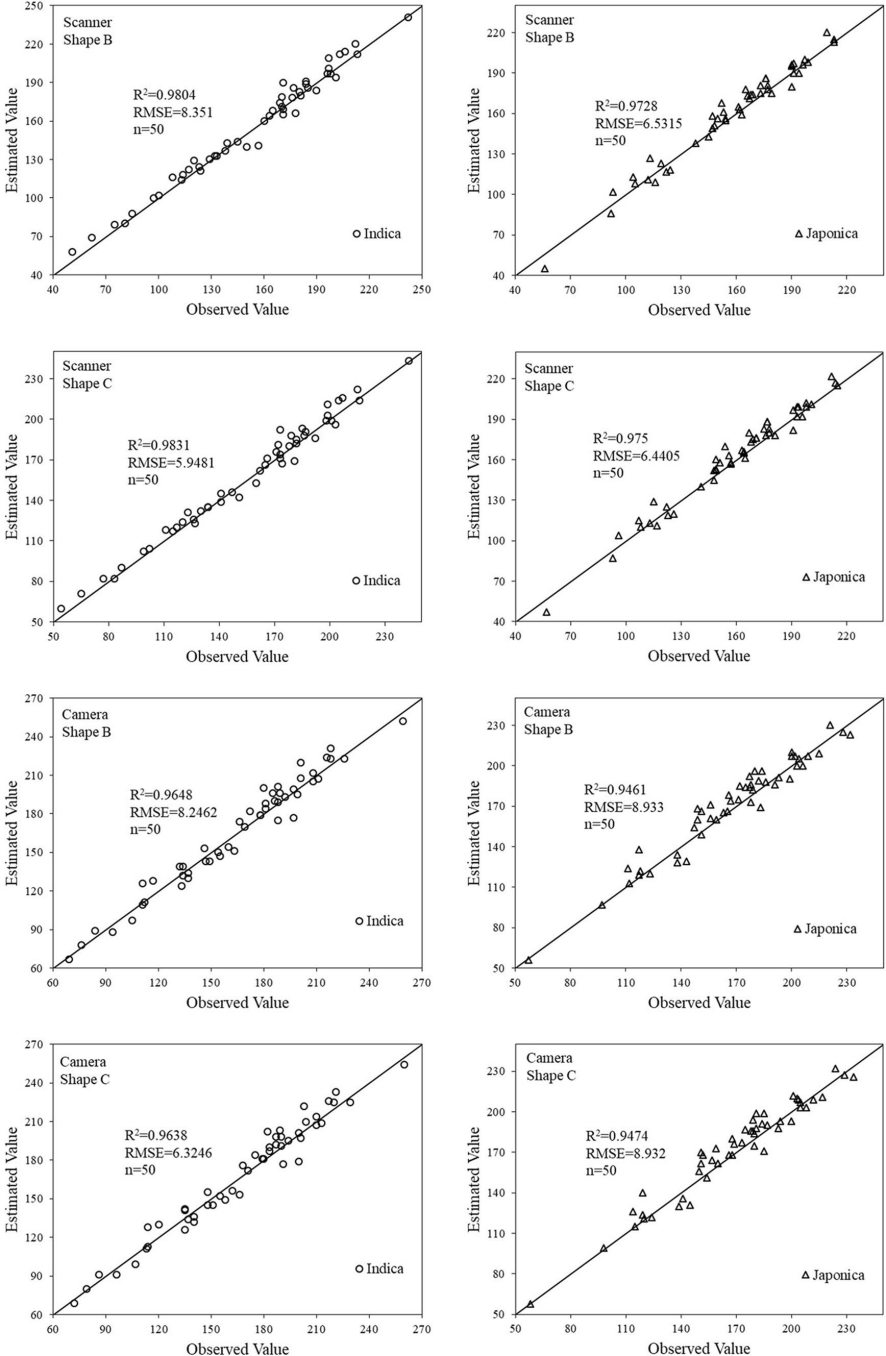
Validation of *CDʹ*-based linear regression model

We validated the *CDʹ*-based linear regression model by comparing the model predictions for grain number with the actual measured number (Fig. 6). With an *R*^2^ = 0.9831 and *RMSE* of 5.9481 for *Indica* rice, and *R*^2^ = 0.975 and *RMSE* of 6.4405 for *Japonica* rice, the Scanner + Shape C method had the best performance in all ways. The Scanner + Shape B also had better performance as compared with the Camera + Shape B and Shape C. Therefore, scanner-acquired Shape C, in combination with *CDʹ*-based linear regression model, provided the most accurate grain counting.

We also constructed a multiple linear regression model based on the three parameters. Table 5 shows the optimal model results obtained after screening. Compared to the univariate linear model (Figs. 5, 6), the multiple linear regression model requires more input factors, however their accuracy has not been greatly improved. Therefore, this study is more inclined to the univariate linear regression model.

**Table 5:** Training and validation of optimal multiple linear regression model

Rice subspecies	Combination method	Training			Validation	
		Models	R^2^	RMSE	R^2^	RMSE
Indica	Scanner + Shape B	GN = 364.93 × CDʹ + 0.70 × Skʹ − 3.90 × Coʹ + 2.801	0.990	4.6732	0.980	6.3254
	Scanner + Shape C	GN = 363.72 × CDʹ + 10.50 × Skʹ − 13.48 × Coʹ + 5.348	0.990	4.6345	0.980	6.3574
	Camera + Shape B	GN = 396.82 × CDʹ − 21.70 × Skʹ − 7.32 × Coʹ + 11.823	0.974	7.6989	0.965	8.3016
	Camera + Shape C	GN = 395.60 × CDʹ − 11.90 × Skʹ − 16.90 × Coʹ + 14.369	0.975	7.5595	0.964	8.3956
Japonica	Scanner + Shape B	GN = 481.49 × CDʹ + 178.22 × Skʹ − 164.85 × Coʹ − 18.485	0.979	6.0957	0.975	6.4714
	Scanner + Shape C	GN = 482.28 × CDʹ + 178.63 × Skʹ − 164.00 × Coʹ − 17.031	0.980	5.9838	0.976	6.4587
	Camera + Shape B	GN = 500.64 × CDʹ + 188.62 × Skʹ − 205.06 × Coʹ − 5.477	0.954	9.1121	0.953	8.5389
	Camera + Shape C	GN = 501.43 × CDʹ + 189.03 × Skʹ − 204.22 × Coʹ − 4.023	0.954	9.0961	0.953	8.5910

### Deep learning model

**Fig. 7:**
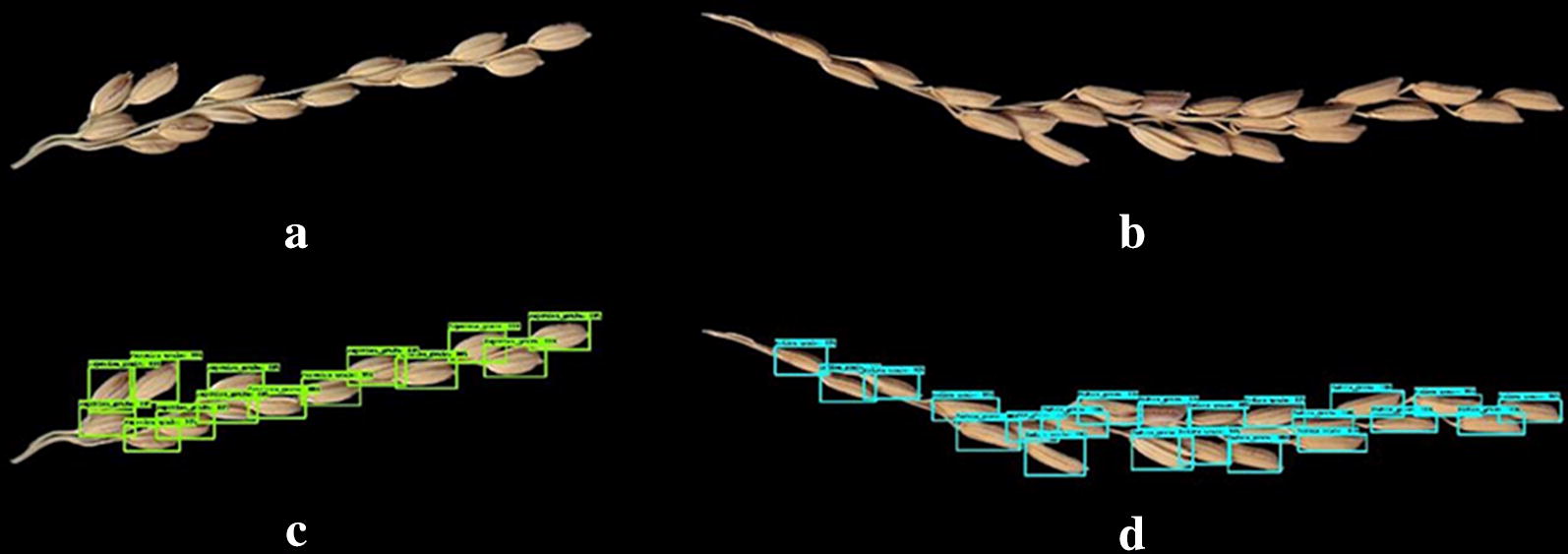
Recognition of grains using deep learning algorithm. **a** Original image of *Japonica* rice. **b** Original image of *Indica* rice. **c** Recognition of *Japonica* rice grains. **d** Recognition of *Indica* rice grains

The deep learning model gave satisfactory performance for grain detection. The grains of both *Indica* and *Japonica* rice were easily detectable (Fig. 7). The counting accuracies of the deep learning models were all > 98%, and were unlikely to be affected by devices and panicle shape (Table 6). Specifically, the Scanner + Shape C method had the highest counting accuracy of 99.38% and a false detection rate of 0%, as well as a very low miss detection rate.

**Table 6:** Grain counting accuracy of the deep learning model

Image acquisition device	Panicle shape	Miss detection rate (%)	False detection rate (%)	Accuracy (%)
Scanner	Shape B	0.79	0	99.21
	Shape C	0.62	0	99.38
Camera	Shape B	1.40	0	98.60
	Shape C	1.02	0	98.98

The results were based on 401 images in validation set

## Discussion

**Fig. 8:**
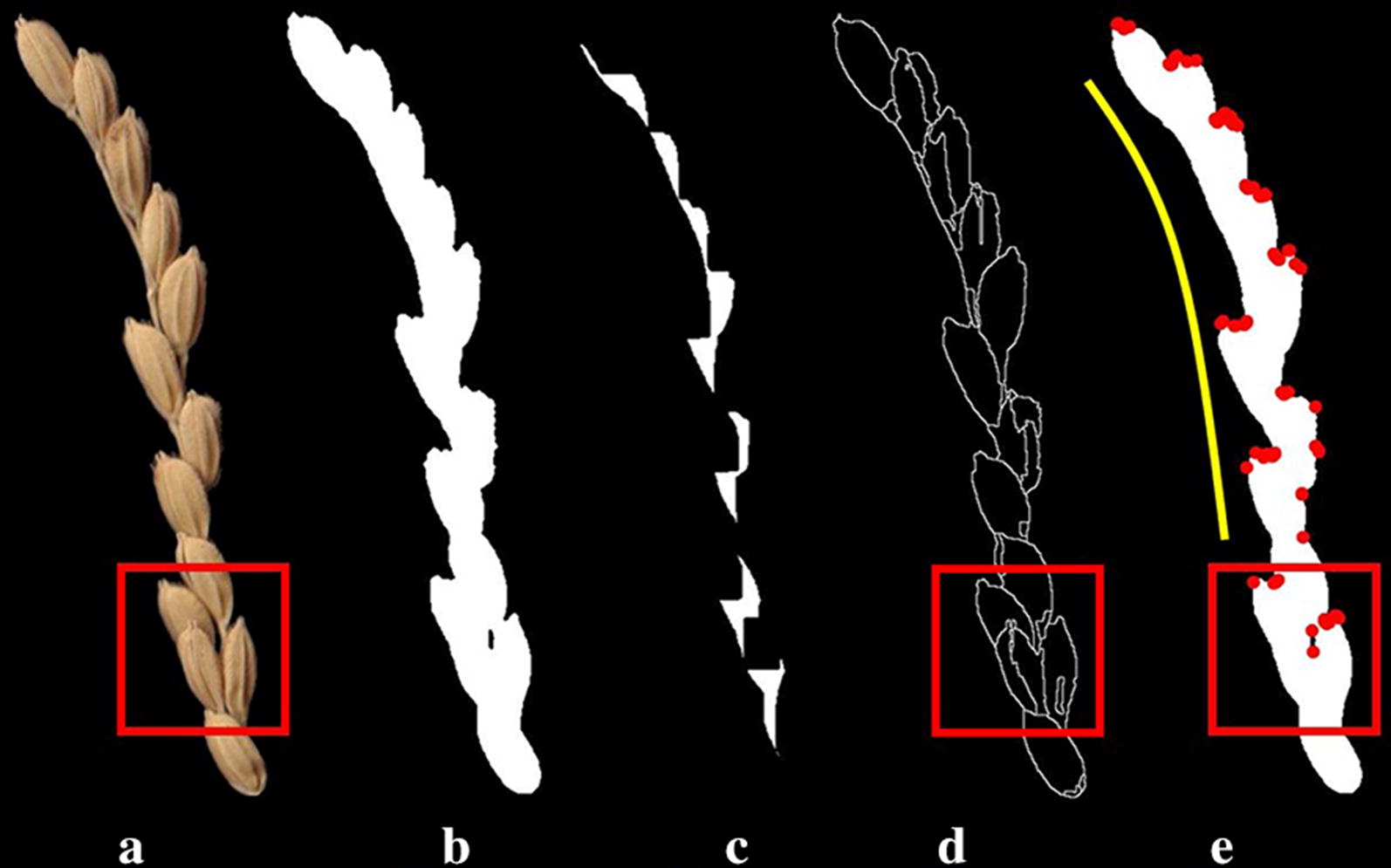
Limitations of traditional image-analysis based grain recognition methods. **a** Original image. **b** Binary image. **c** Dilation and erosion operation results. **d** Improved watershed method. **e** Corner detection and feature matching method

In this study, we not only developed a linear regression model and a deep learning model to count the grain number per rice panicle, but also tested the grain counting efficiency of traditional image-based counting methods. Traditional grain counting methods rely primarily on the separation of touching grains, and mainly algorithms include the dilation and erosion method [25], the improved watershed algorithm [26], and feature point matching method [27, 28]. The commonly encountered problems of these traditional methods are shown in Fig. 8. The dilation and erosion method often fail to separate touching kernels (Fig. 8c). The improved watershed algorithm was able to detect the kernel edges, but the kernel surface was over-separated and could not account kernel number accurately (Fig. 8d). The corner detection and feature matching methods (Fig. 8e) had an unsymmetrical detection capability and no corner point was detected on the yellow line side, while more corners were detected on the other side. Therefore, we do not believe that this method can provide satisfactory separation.

The removal of stems during image preprocessing improves the counting accuracy. We compared the model performance in the presence and absence of stems using accuracy as comparison criteria (Table 7). Both the linear model and the deep learning model improved counting accuracy after stem removal, with a maximum improvement of 3%. The deep learning model had significantly higher counting accuracy compared to the linear model, which exhibited as high as a 10% accuracy difference between different rice types. Meanwhile, the deep learning model had a similar performance regardless of rice types. These results demonstrated that the impact of rice type on the model performance was significant for the linear model, but had less of an effect on the deep learning model.

**Table 7:** The effect of stems on the counting accuracy of different models

Model	Rice subspecies type	Stems	Accuracy (%)
Linear regression model	*Indica* rice	Yes	96.95
		No	97.84
	*Japonica* rice	Yes	95.48
		No	96.43
	*Indica* + *Japonica* rice	Yes	84.86
		No	87.56
Deep learning model	*Indica* rice	Yes	98.84
		No	99.06
	*Japonica* rice	Yes	99.36
		No	99.52
	*Indica* + *Japonica* rice	Yes	99.16
		Yes	99.38

The results in this table were from scanner acquired images and the Shape C panicles

**Fig. 9:**
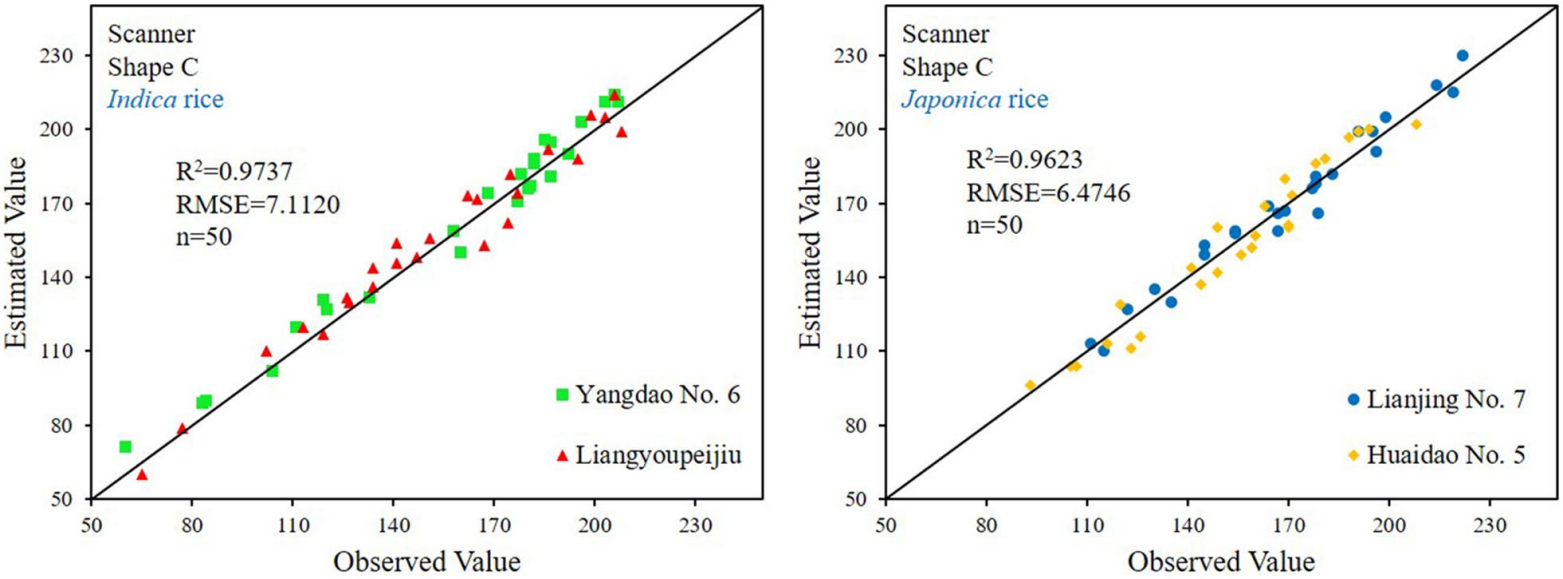
Robustness analysis of linear regression models

To further validate the robustness of linear regression models, we used two varieties of *Indica* rice (Yangdao No. 6 and Liangyoupeijiu) and two varieties of *Japonica* (Lianjing No. 7 and Huaidao No. 5) as new materials to analyze subspecies varieties. Take the Scanner + Shape C method as an example, and the verification results are shown in Fig. 9. The R^2^ and RMSE of the *Indica* rice model were 0.9737 and 7.1120 respectively. The R^2^ and RMSE of the *Japonica* rice model were 0.9623 and 6.4746 respectively. Compared with the results of Fig. 6, there is a slight decrease. So, the robustness of linear regression models is greatly affected by the shape of the panicle and grain, and is less affected by the size of the panicle and grain. The shape is mainly controlled by the subspecies, and the size is controlled by cultivation measures such as variety, density and nitrogen application rate.

**Fig. 10:**
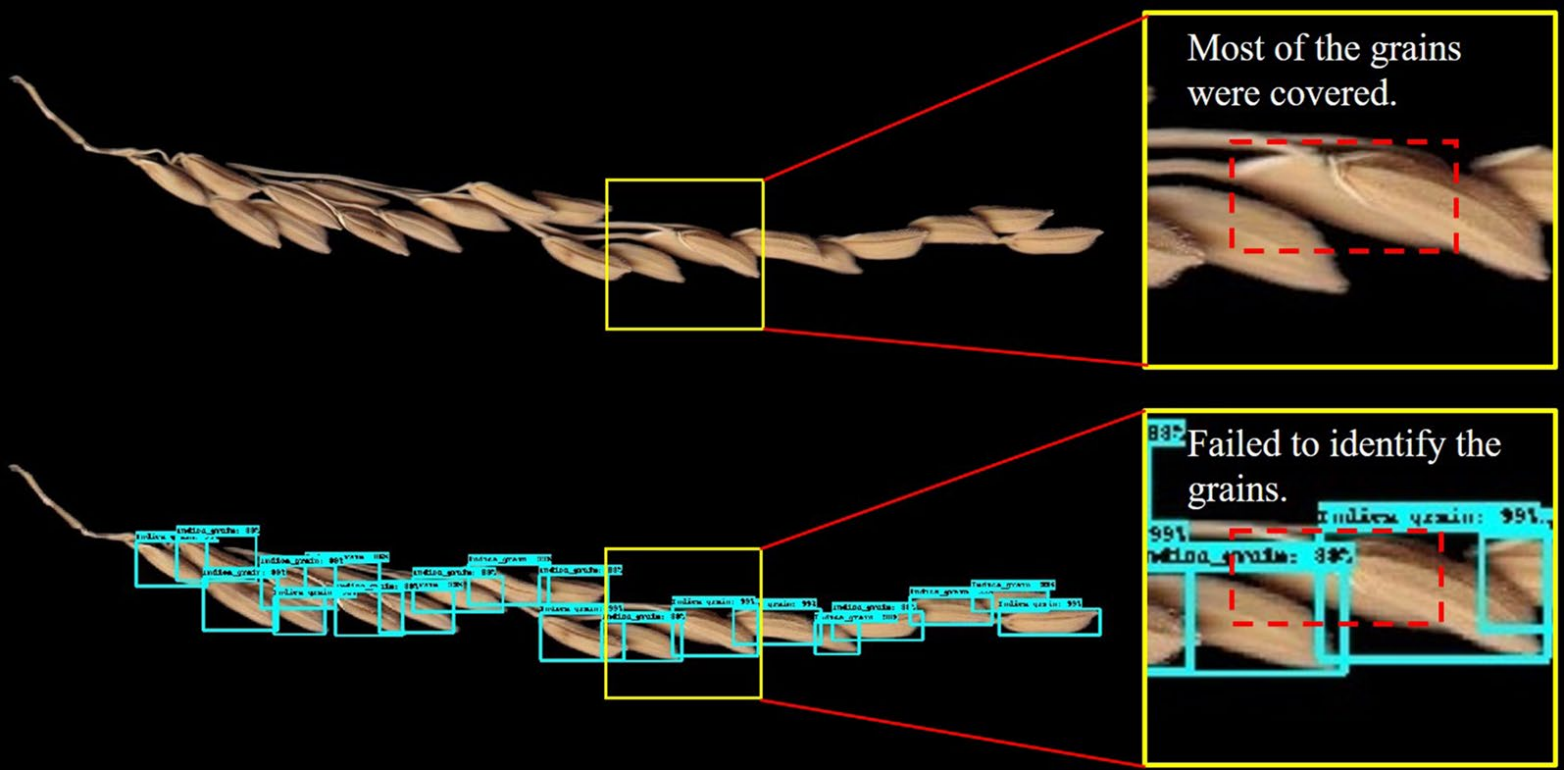
Limitation of the deep learning model in *Indica* rice grain counting

The deep learning model did have a slightly higher performance for the *Japonica* rice than the *Indica* rice. We analyzed images from test set that had a low counting accuracy (Fig. 10). The *Indica* rice grains were long and slender, which make them more likely to form a cluster and consequently, can’t be detected as effectively by the deep learning model. Therefore, the counting accuracy of *Indica* rice is lower than that of *Japonica* rice regardless of model.

When using the image-based algorithm, the running time of the model was also an important factor. We took 20 panicle images as a group and calculated the time spent. Repeat 5 times and take the average as results (Table 8). The time needed for counting was twofold: the time needed for image acquisition and the time needed to run the algorithm. The Shape A image acquisition time was the fastest among these conditions. However, the results in Table 4 showed that the accuracy of using Shape A images was low. The use of X-ray technique or three-dimension (3D) image will have a better performance on Shape A. Charytanowicz et al. [29] used X-ray images to evaluate geometric features for wheat grain classification. However, X-ray equipment is expensive. Long-term acceptance of X-rays can cause a lot of damage to human body. Generally, the accuracy of the 3D scanner is high, which is suitable for obtaining large objects such as forest trees [30]. For small targets such as rice, expensive higher precision instruments are required. In addition, the acquisition and processing of 3D images may take more time. In summary, we take Shape B and Shape C as research objects rather than Shape A.

**Table 8:** Time needed for each work

Image acquisition device	Panicle shape	Time used
Scanner	Shape A	2 m 30 s
	Shape B	5 m 40 s
	Shape C	4 m 46 s
Camera	Shape A	40 s
	Shape B	3 m 20 s
	Shape C	2 m 26 s

Counting method	Time used	
Manual counting	21 m 40 s	
Linear regression model	3 s	
Deep learning model	2 m 20 s	

The time needed to acquire images using a digital camera was 50% of that using scanner, and the time needed for Shape C was shorter than Shape B. During the time experiment, Shape B needs to carefully separate the intertwined branches, and the violent operation will cause the individual grains to fall off and affect the subsequent treatment. Shape C can be separated from the stem by cutting a knife. Therefore, Shape B takes more time than Shape C. When running the linear model, most of the time was used for image processing and for the extraction of parameters. Meanwhile, when running deep learning model, most time was spent on parameter loading. The linear regression model required significantly less time than the deep learning model due to the fact that the deep learning model must load millions of parameters and involves large amount of data execution. The Scanner + Shape B + Deep Learning method takes 8 min, which is only about one-third of the manual counting time. The brain power expended by manual counting was not included yet. Thus, using multiple sets of high-performance graphics processing unit (GPU) could significantly accelerate data execution [31]. In addition, model compression or the establishment of a simpler deep learning model could also reduce the model running time [32].

## Conclusion

In summary, we established two models to count the grain number per panicle, a linear regression model and a deep learning model, which had a counting accuracy greater than 96% and 99%, respectively. However, the deep learning model required more time than the linear regression model. If we consider the time cost, linear regression model is recommended for counting the rice grain number per panicle. Otherwise, the deep learning model would be best to optimizing accuracy. We believe our high-throughput and rapid method for counting the number of rice grains per panicle is a useful tool for rice phenomics research.

## Data Availability

All data analyzed during this study are presented in this published article.
